# Gluteal Compartment Syndrome following Vascular and Neurological Injuries

**DOI:** 10.1155/2014/869139

**Published:** 2014-02-23

**Authors:** Mahmoud A. Hafez, Moustafa Radwan

**Affiliations:** The Orthopaedic Department, October 6 University, Giza 11787, Egypt

## Abstract

Gluteal compartment syndrome is a serious but rare condition that has recently been increasingly reported in literature. This report presents two cases that followed neurological and vascular injuries (first case: an injury of the superior gluteal artery; second case: neurological injury bilaterally). A high index of suspicion and attention is needed to early diagnose this condition due to the possibility of developing serious and potentially fatal complications and difficulty in management.

## 1. Introduction

Gluteal compartment syndrome is a serious condition that can be overlooked. Although it is a rare phenomenon, it has recently been increasingly reported in literature. This case series includes 2 case reports of life-threatening gluteal compartment syndrome due to neurological and vascular injuries. One case was due to an injury of the superior gluteal artery and the other case was due to neurological injury bilaterally, which led to 6 episodes of compartment syndrome involving buttocks, thighs, and legs that was considered life threatening too.

Surgeons are not familiar with the nature of gluteal compartment syndrome following neurological or vascular injuries. A high index of suspicion is needed to early diagnose this condition, and attention should be paid for the possible serious and potentially fatal complications as well as the difficulty in management.

## 2. Case 1

**Figure 1:**
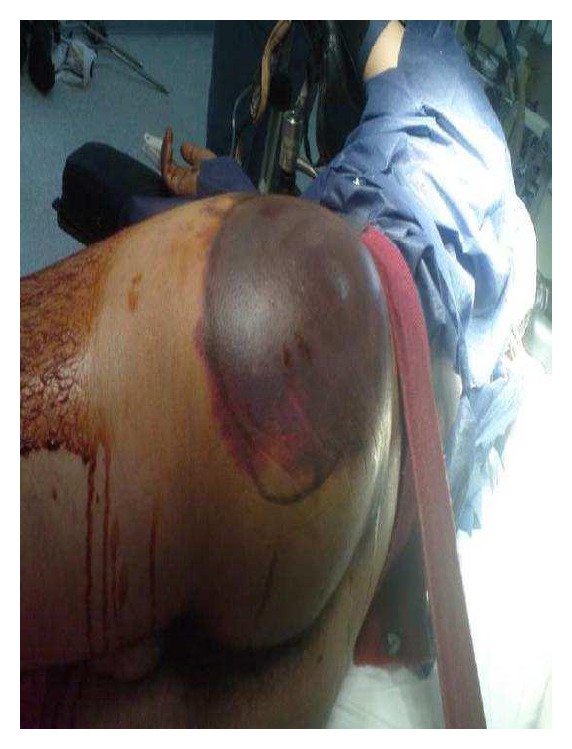
Severe hematoma of the buttock following a gluteal compartment syndrome due to a vascular injury.

A 24-year-old male was involved in a car accident and admitted to the general surgery department as a query abdominal injury. He was shocked with hemoglobin of 7.2 mg/dL, but clinical and radiological investigations (US and CT) showed no evidence of abdominal injury and no internal bleeding apart from retroperitoneal hematoma and tense left buttock with severe hematoma that was thought to be a contusion (Figure 1). The patient was kept under observation with a provisional diagnosis of retroperitoneal hematoma. Later, he developed sensory and motor loss in his left foot that raised the suspicion of gluteal compartment syndrome. Afterward, he was transferred from general surgery to the orthopedic department, where the patient was further examined, to find out hip swelling (firm and tender swelling). This finding, together with the sensory and motor loss, immediately urged us to take the patient to the operating theater, so fasciotomy of gluteal compartment was done. A massive bleeding happened once the fasciotomy was performed due to the loss of the tamponade effect. There was a great difficulty to control the bleeding as it originated from deep down in the pelvis. The patient lost more than 2 liters in few minutes to an extent that threatened his life. Extensive packing was used until a vascular surgeon arrived and used clips to control the bleeding from the superior gluteal artery that was injured deep inside the pelvis. Debridement of necrotic muscles was done and a drain was inserted. The patient received massive blood transfusion and survived this episode. Two days later, he had a second look and debridement but no further bleeding was there. He developed superficial skin infection that was managed by antibiotics. His sensory and motor functions improved afterwards.

## 3. Case 2

A 44-year-old woman was found unconscious at home 24 hours after falling and hitting her head. She was discovered in a kneeling position with bruises in the occipital area. She had a tense left calf and a known history of alcoholic liver disease. CT of the head showed a right acute subdural hematoma. The pressures of the left leg compartments were raised from 80 to 120 mmHg (BP 150/70 mmHg). Her creatinine phosphokinase was 41800, WCC15.5, and pH 7.32. Urine analysis revealed hematuria and proteinuria. The diagnosis of crush injury due to left leg compartment syndrome was made. After initial resuscitation and induction of alkaline diuresis, she was taken to the operating room for craniotomy and left calf fasciotomy. On review at 12 hours, she was noted to have tense bilateral buttocks, thighs, and right leg compartments with increased intracompartmental pressure of 50–70 mmHg (BP132/60 mmHg). The diagnosis of compartment syndrome was established as we assumed that the kneeling position resulted in elevated intramuscular pressure in the buttocks, both thighs, and right leg. The kneeling position resulted in venous outflow obstruction that had, together with the head bruise, predisposed the patient to develop this acute compartment syndrome.

The patient underwent immediate decompression of all these compartments. She survived but she lost all of her left calf muscles (except the peroneal), as well as her right medius, minimus, and part of the gluteus maximus.

## 4. Discussion

Compartment syndrome of the gluteal region was reported following trauma [1], drug-induced coma [2], Ehlers-Danlos syndrome [3], and positioning during surgical procedures [4], and after vascular injury [5]. Gluteal compartment syndrome due to traumatic injury of the superior gluteal artery was reported following a low-energy injury [6], simple hip dislocation [7], bone marrow biopsy [8], intramuscular gluteal injection [9], iliac bone grafting [10], and robotic-assisted prostatectomy [11]. Literature review revealed few reports associated with vascular and neurological injuries. In Taylor et al. case, exploring a hematoma in the gluteal region surgically has shown a ruptured gluteal artery trunk which was clotted off. However, in Case 1, a male patient similarly had a ruptured superior gluteal artery trunk but the vessel was not clotted off; therefore, bleeding was very difficult and resulted in a near-fatal situation. No reports have shown that neurological trauma has resulted in gluteal compartment syndrome (GCS).

However, in our work, we indicated that neurological traumas cause GCS. Thus, a female patient was found in kneeling position due to neurological trauma. This resulted in a coma that disabled her from changing her position for 2 days. This kneeling position resulted in compartment syndrome in 6 compartments; gluteal, thigh, and calf compartments bilaterally. This has not been found in literature.

In this case series, Case 1 had a massive bleeding due to injury of the superior gluteal artery with no fractures. The tight compartment was the limiting factor for the continued bleeding and worked as a tamponade. On the other hand, the fasciotomy and the release of the gluteal compartment, which is the standard treatment, had a serious drawback of detamponading the compartment and the loss of hemostasis leading to massive bleeding. The cut in the superior gluteal artery was deep in the pelvis and neither visible nor easily accessible. The packing and compression over the bleeding vessel until a vascular surgeon arrived and clamped the bleeding vessel have helped the patient to survive.

In the bilateral case (Case 2), the occurrence of compartment syndrome in six large compartments (both buttocks, both thighs, and both legs) resulted in massive rhabdomyolysis and crush syndrome, which was life threatening as well. The early detection of one compartment syndrome in this patient and the close observation led to the discovery of the compartment syndrome in the other five regions. The prompt release and fasciotomy helped the patient to survive this potentially fatal condition.

Diagnosing the compartment syndrome in Case 2 was overlooked on the patient admission to the hospital. This diagnosis requires high suspicion, skillful clinician, and profound examination as the condition is very rare. GCS can be prevented by avoiding its causes (especially posttraumatic) by early diagnosis and following strict instructions to the patients after traumas or surgical procedures.

## 5. Conclusion

A high index of suspicion is needed to early diagnose gluteal compartment syndrome following neurological and vascular injuries. Attention should be paid to the possible serious complications and the difficult management of such condition.
